# Pica in a Patient With Decompensated Schizophrenia

**DOI:** 10.7759/cureus.17964

**Published:** 2021-09-14

**Authors:** Xiao Xiong You, Baris Olten, Kunal Gandhi, Saral Desai, Adela Gerolemou

**Affiliations:** 1 Department of Psychiatry, Brookdale University Hospital Medical Center, Brooklyn, USA

**Keywords:** psychiatry, psychosis, eating disorders, schizophrenia, pica

## Abstract

Although pica is commonly associated with nutritional deficiencies, it is also observed in psychiatric disorders such as obsessive-compulsive disorder and less commonly in schizophrenia. We describe a case of pica in a 34-year-old male with decompensated schizophrenia. Emergency medical services brought the patient from a state facility as he was scavenging and eating foreign objects. Upon initial evaluation, no notable nutritional deficiencies were noted. After surgical removal of foreign objects, he was started on antipsychotics. His pica was determined to be due to his active psychosis involving delusions, disorganized thought processes, and loosening of associations. His psychosis improved on paliperidone intramuscular injection and oral olanzapine, which coincided with reduction and resolution of pica. Our case highlights the need to understand further the exact psychopathology of pica that may not be limited to nutritional deficiencies.

## Introduction

Schizophrenia is one of the most disabling medical disorders classified by the WHO global burden of disease 2017 report, affecting 20 million people worldwide [1]. Schizophrenia spectrum disorders are defined by abnormalities in one or more of the following domains such as perception, emotion, cognition, and thoughts, and can present with delusions, hallucinations, disorganized thinking (speech), grossly disorganized or abnormal motor behavior (including catatonia), and negative symptoms including social withdrawal, apathy, and anhedonia [2].

Pica is an eating disorder that consists of the persistent eating of non-nutritive or non-food substances over a period of at least one month [1]. During this time period, it also must occur at an age where eating said non-food substances would be considered developmentally inappropriate. It must not be attributable to a culturally sanctioned practice or in the context of another mental disorder (such as intellectual disability) or a medical condition (e.g., pregnancy). Pica can involve ingestion of any number of non-food substances and tends to range based on age and availability. Typically, the most commonly ingested substances are paper, soap, hair, nails, and ice. It is commonly associated with iron deficiency anemia and chronic malnutrition [3]. It is well documented that patients who suffer from chronic malnutrition often present with pica. In these instances, the ingestion of non-food substances is an attempt to correct significant nutritional deficiencies [3]. Published case reports of pica associated with psychosis are rare.

In this case report, we present a case study of a male patient transferred from the general surgery floor to the inpatient psychiatric unit due to an acute exacerbation of his chronic schizophrenia. This patient was found to have ingested multiple foreign objects, including coins, nails, paper, and glass. In this case report, we will discuss how pica can be a manifestation of thought disorder and delusions.

## Case presentation

A 34-year-old male with a past psychiatric history of schizophrenia and cocaine use disorder and medical history of asthma was brought to the emergency room by emergency medical services (EMS) from a state-run transitional housing residence as he was found consuming various objects around the residential grounds. The patient has had numerous previous abdominal surgeries due to ingestion of foreign bodies. He was domiciled in a state-run transitional housing residence for approximately one year prior to his current hospital presentation. As per facility staff, the patient was scavenging around the residence, eating garbage and other foreign objects. He has no neurological history, and he was hospitalized multiple times in the past for decompensated schizophrenia and resultant abdominal surgeries due to pica during his decompensation. His vital signs were within the reference range and he was afebrile. Initial lab results showed hemoglobin of 14.4 g/dl, mean corpuscular volume (MCV) of 92.4 fL, mean corpuscular hemoglobin (MCH) of 29.5 pg, mean corpuscular hemoglobin concentration (MCHC) of 31.9 g/dl, and red cell distribution width (RDW) of 15%. His urine toxicology results were negative for any illicit substances. Full medical workup conducted in the emergency room did not show any acute metabolic derangements. Other tests to rule out an organic cause of altered mental statuses such as non-contrast computed tomography scan of the head and rapid plasma reagin (RPR) were negative as well. Although the patient did not receive MRI during this admission, MRI done during his previous admission with similar pica behavior was negative.

**Figure 1:**
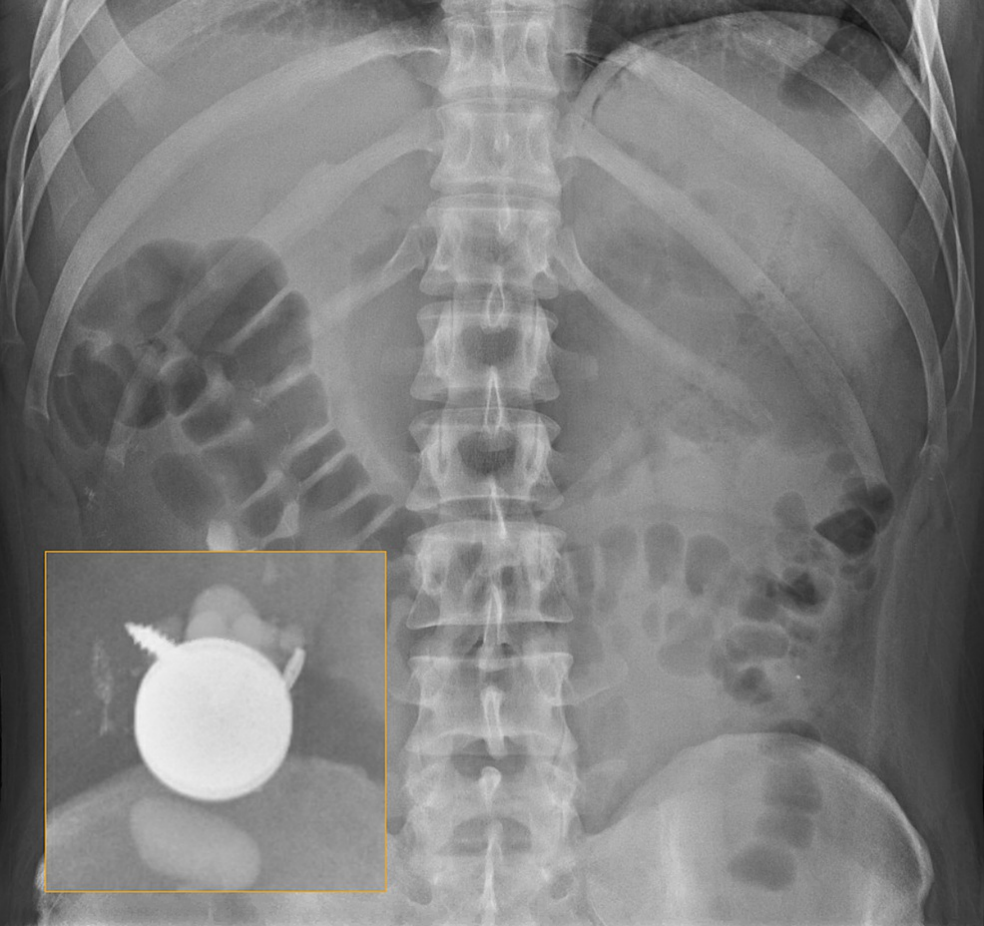
X-ray of the abdomen. Foreign objects identified in the right lower quadrant of the abdomen.

**Figure 2:**
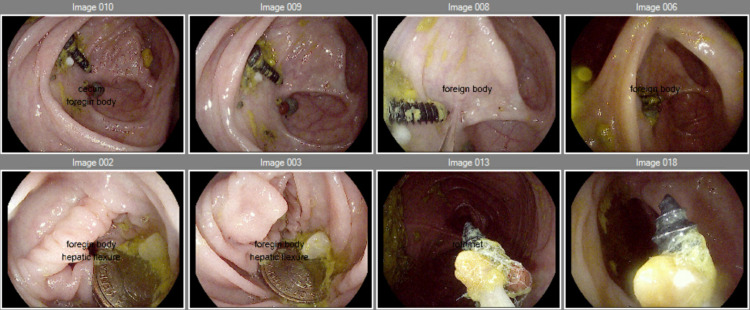
Colonoscopy procedure. Colonoscopy images showing foreign objects identified as screws and coins in the cecum.

His home medications included Abilify 20 mg orally (PO) daily for his schizophrenia. As per facility staff, he was minimally adherent to his medications. Upon arrival in the emergency room, the patient reported to the emergency department (ED) physician that he had been eating things like hair conditioner, baby oil, pebbles, coins, glass, and screws. Had poor insight into his psychiatric illness and displayed disorganized thought processes and behavior. He reported vomiting for the past day. Psychiatry was initially consulted for medication recommendation and management of his schizophrenia as the patient appeared grossly psychotic, guarded, and paranoid. He was then admitted to the surgical service for the removal of ingested foreign objects. An abdominal X-ray was obtained (Figure 1). Laparoscopic removal of foreign bodies was performed (Figure 2). The psychiatry consultation service recommended starting the patient on Risperdal 1 mg PO in the morning and 2 mg PO at bedtime for his acute psychotic symptoms.

Subsequently, upon medical clearance, the patient was transferred to the inpatient psychiatry unit for further stabilization. His medication was then increased to Risperdal 2 mg PO twice a day. The patient's psychosis slightly improved with risperidone, but he continued to exhibit disorganized thought processes, illogical thought content and continued to ingest objects on the unit. The ultimate plan was to start the patient on long-acting injectable antipsychotics to reduce relapse following discharge from the hospital. Paliperidone 234 mg intramuscular (IM) was given to the patient. Additionally, olanzapine 10 mg once a day was also started as a second antipsychotic as an augmentation to manage his psychotic symptoms as monotherapy was having a minimal effect.

Olanzapine was titrated up to 20 mg once a day, and improvement in overall psychosis followed. He started to exhibit more organized thought processes with logical associations. In addition, he was found to be consuming appropriate meals and not found scavenging for non-food substances. Oral risperidone was discontinued after the administration of the maintenance dose of intramuscular paliperidone 156 mg. The patient continued to improve. His thought process was notably more linear and logical, and this improvement of his disorganized thought process coincided with the cessation of his desire for consumption of non-food substances. Upon re-evaluation of the patient with his case manager before discharge, the patient appeared to be at his baseline and he was discharged to his residence with outpatient follow-up in the community.

## Discussion

Abnormal feeding behaviors observed in schizophrenic patients, including refusal to eat and, less commonly, pica, are often correlated to positive symptoms and dimensions of their psychopathology [4,5]. Pica may also be a manifestation of repetitive behaviors seen in schizophrenia [6]. However, abnormal feeding behavior and poor functional status in chronic schizophrenic patients might also lead to nutritional deficiencies, potentially leading to pica [7].

While some authors postulate that it might be a symptom of obsessive-compulsive disorder, which is highly comorbid with schizophrenia [8,9], Others suggest that pica may be induced by antipsychotic medications [10]. Demonstration of pica in patients with schizophrenia goes back in literature as far as Bleuler, who attributed abnormal eating behaviors to delusional ideas [11]. Although pica is relatively rare in schizophrenic patients, when present, undiagnosed pica can lead to hazardous medical complications such as metal poisoning, intestinal obstruction, intestinal bleeding, intestinal perforation, and electrolyte disturbances [12,13]. 

Our patient did not endorse any obsessions and/or compulsions besides persistent eating behavior of non-edible substances. He denied any urge preceding the ingestion of foreign objects. When asked about what goes through his mind before eating non-edible substances, the patient reported that he eats metal because he always desired to be a cop. When he was asked to elaborate, he reported that cops carry a metal gun. It was clear to us that our patient was suffering from a severe form of thought disorder. We postulate that pica was not a compulsion in our case but rather resulted from loose associations and bizarre delusions of his decompensated schizophrenia. Our patient’s pica also could not be attributed to iron deficiency anemia as the patient's hemoglobin levels, including red blood cell indices, were within reference range upon presentation.

Our patient’s improvement of psychosis with antipsychotics coincided with the decline of the frequency of pica. We hypothesize that as our patient’s delusions and disorganized thoughts improved, he grasped the bizarre nature of his behavior. After improving psychosis and pica, the patient was asked again why he was eating the non-edible substances. He reported being embarrassed about eating these substances and said he could not think of any logical reason for eating them. We found it futile to do additional testing to detect any nutritional deficiencies as the patient's pica successfully resolved with the improvement of his psychosis.

In conclusion, we believe it is important to keep pica in the differential diagnosis in patients with schizophrenia presenting gastrointestinal symptoms such as bleeding, abdominal pain, persistent nausea, and vomiting. Retrospective investigation of cohorts of patients with schizophrenia and pica could give us the relative frequency of this rare condition and help us understand its etiology.

## Conclusions

Pica, although often associated with nutritional deficiencies, can manifest as part of disorganized thought processes and behavior in acutely psychotic schizophrenic patients. Pica can lead to severe complications in schizophrenic patients, especially when not diagnosed early during the clinical course. Further research is needed to explore the exact pathophysiology of pica and its potential association with schizophrenia.
